# A qualitative assessment of alternative eradication strategies for African swine fever in the Dominican Republic

**DOI:** 10.3389/fvets.2022.1054271

**Published:** 2022-11-17

**Authors:** Rachel Schambow, Raysa Reyes, Jose Morales, Alan Diaz, Andres M. Perez

**Affiliations:** ^1^Center for Animal Health and Food Safety, University of Minnesota, St. Paul, MN, United States; ^2^Department of Veterinary Population Medicine, College of Veterinary Medicine, University of Minnesota, St. Paul, MN, United States; ^3^Instituto del Estudio de las Enfermedades Zoonóticas, Universidad Autónoma de Santo Domingo, Santo Domingo, Dominican Republic

**Keywords:** SWOT, African swine fever, disease control, Dominican Republic, swine, pig, qualitative

## Abstract

Since its recent detection in July 2021, the reintroduction of African swine fever (ASF) in the Dominican Republic (DR) has generated much discourse on various measures for its effective control. Strategies range from complete depopulation of the swine population, as was done in 1978, to a system of passive surveillance with endemicity, with many in-between. Currently, ASF-decision makers need a peer evaluation and comparison and contrast of these potential strategies that incorporates both private and public perspectives. To achieve this, we used strengths, weaknesses, opportunities, and threats (SWOT) analysis to evaluate three different theoretical ASF control scenarios with the aim of contributing evaluations of alternatives strategies to mitigate the epidemic's impact. These included total depopulation of all pigs in the DR, partial depopulation, and continuation of current control measures. Relevant experts from the DR private swine industry were identified through “snowball sampling” techniques. Five experts completed the SWOT questionnaire and additional questions considering aspects of financial cost, social impact, feasibility, animal welfare, and regional policy. The summarized responses were presented to the full group of experts initially nominated for final review and later to representatives of the DR government. The SWOT analysis highlighted that although there are certain benefits associated with each of the proposed strategies, there are also important drawbacks and disadvantages for all. This analysis is a tool for facilitating cooperating between the private-public industries, and ultimately it supports the development of strategies that will reduce ASF burden in the DR in a way suitable for all relevant stakeholders.

## Introduction

African swine fever (ASF) is a reportable hemorrhagic disease of porcine caused by a large, double stranded, DNA arbovirus from the Asfiviridae family, referred to as the African swine fever virus (ASFV) (1, 2). ASFV only affects swine, including domestic pigs, wild boar, African wild warthogs, and other wild suids (1, 3). ASF was first reported in 1921 in Africa, caused widespread outbreaks in the Iberian Peninsula in 1957 and 1960, and spread to the Caribbean and South America, including the Dominican Republic (DR), in the 1970's (3, 4). After immense effort, it was eventually eradicated outside of Africa in all regions but the Italian island of Sardinia. Outbreaks associated with ASF's most recent detection in Georgia in 2007 are still ongoing and represent a major threat to the swine population globally (5, 6). Since this latest detection in Europe, ASF has spread throughout Russia, China, southeast Asia, central Europe, and into the Caribbean. At the time of writing this manuscript, June 2022, it was most recently detected in domestic pigs in Germany near its southwestern border, in wild boar and domestic pigs around Rome, and in Nepal (7).

ASF is difficult to control for many reasons, including its long-term survival across wide pH and temperature ranges, spread through infected pork products, fomites, soft ticks, and wild boar, and the lack of effective treatments or a widespread, commercially available vaccine (8–11). ASF-affected countries have applied different control measures with varying levels of success. For domestic pig management, those control measures typically include components of biosecurity, surveillance, quarantine, epidemiological investigation, movement controls, contact tracing, and depopulation of infected farms (12). In some circumstances, control efforts have been successful in limiting disease spread or supporting ASF eradication (13). Since 1995, the ASF Genotype I strain initially responsible for the outbreaks in Europe and the Americas has remained confined to Sardinia, with no known outbreaks emerging from the island (5, 14). In the current outbreak, only the Czech Republic and Belgium have regained ASF-free status. Both countries only reported cases in wild boar, never in domestic pigs. In 2014, the Czech Republic enacted early passive surveillance of all dead pigs within the country. They first detected ASF in wild boar in June 2017 (13). Their successful eradication plan incorporated risk-based zoning, intensive hunting, and nationwide surveillance and carcass removal. The epidemiological context in each country necessitates a country-specific approach. In some regions, wild boar and feral pigs are highly important drivers of the disease, while in others they are absent. The human social structure, pig sector structure, and economic constraints of each affected country are also highly important to ASF control (15).

In July 2021, ASF was reported in the Dominican Republic (DR) and later officially confirmed in Haiti in September (7, 16, 17). Reports to WOAH (formerly OIE) suggest that the initial introduction into the DR may have occurred as early as April; it is unclear when it was introduced into Haiti (18). This new jump to Hispaniola presents an increased risk for ASF introduction into other countries of the Americas and is causing severe hardship for the DR swine industry. Though it is difficult to estimate the number of pigs raised in non-commercial farms, there are approximately 1–2 million pigs in the DR, of which about 62,000 sows are raised in commercial units (19). Since recognition of ASF in the DR in July 2021, private and public stakeholders have attempted to control disease spread (20). In its first encounter with ASF in 1978, the DR used its military to implement a full-scale eradication of its pig population (13, 21). Initial contingency plans for the current outbreak seek to detect and eradicate ASF-positive herds, many of which came from backyard farm operations, with compensation for depopulated farms (22). Due to lack of infrastructure, samples were initially being tested and confirmed at the United States Department of Agriculture's (USDA) Foreign Animal Disease Diagnostic Laboratory (FADDL) on Plum Island. In the fall of 2021, official PCR testing became accessible in the DR's Central Veterinary Laboratory (LAVECEN), significantly decreasing test turnaround time (23). Ongoing attempts to control disease spread have included the use of passive surveillance by farmers and producers, with animal health officials investigating suspicious reports and applying testing, quarantine, and movement controls where appropriate. As of October 2021, reports suggest the government has paid over 530 million Dominican pesos (~9.67 million USD) in compensation for more than 74,000 pigs nationwide, representing approximately 3.7–7.4% of the population (19, 24). The Ministry of Agriculture has also promoted repopulation of backyard farms with non-swine species, such as goats, chickens, and cows (24).

**Figure 1:**
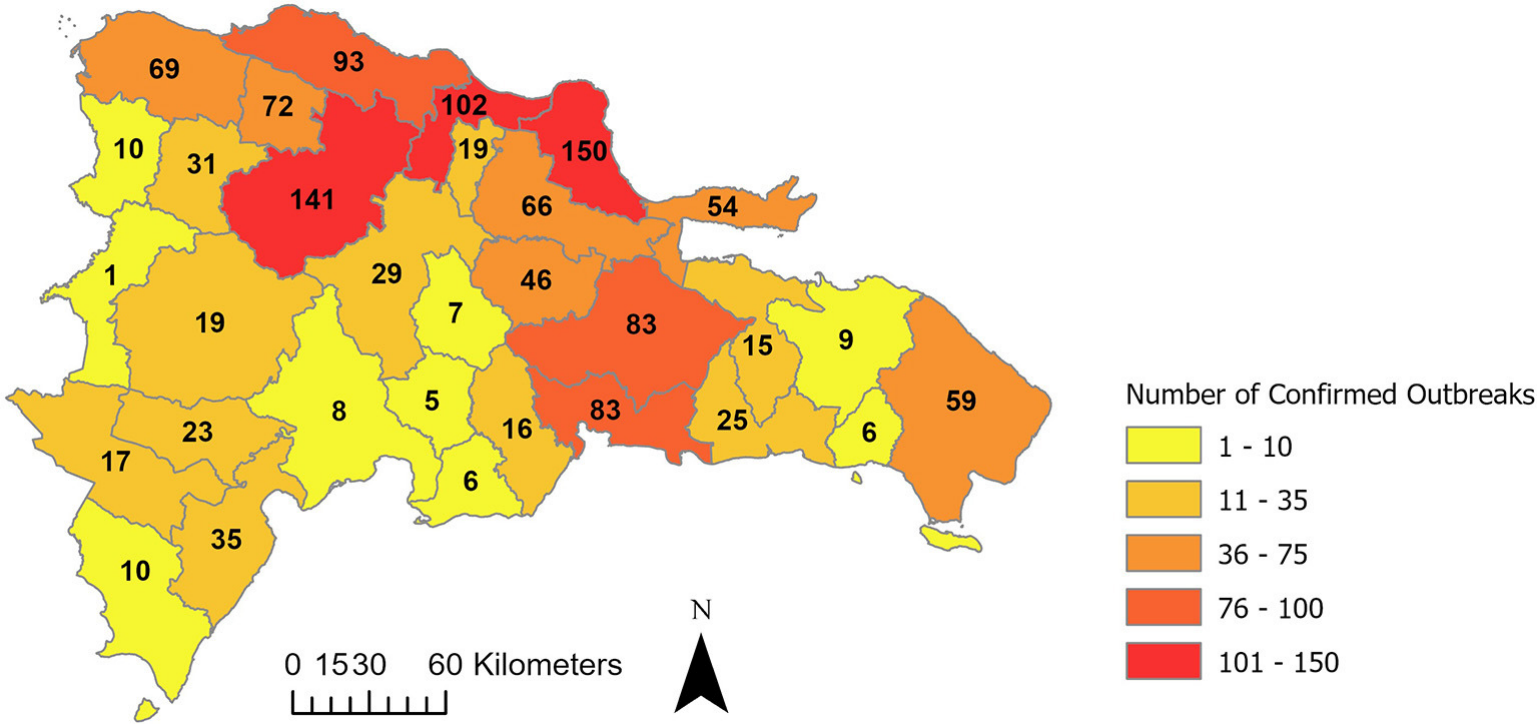
Cumulative number of confirmed outbreaks of African swine fever by province in the Dominican Republic from May 22, 2021–May 28, 2022. Data from Gonzales and Romero (25).

Despite these efforts, outbreaks continue to occur throughout the DR, and concerns from public and private stakeholders that the disease will become endemic are growing. The lack of traceability of pig premises locations and movements, especially those of small and backyard producers, and limited government resources, continue to constrain the implementation of ASF mitigations. Challenges associated with the disease, and demographic, social, and political factors, have limited the success of containing ASF spread in the DR. As of June 2022, a total of 1,310 outbreaks distributed in 30 provinces have been confirmed through laboratory diagnosis (24), (Figure 1).

The success of measures like biosecurity, surveillance, reporting, quarantine, and movement restrictions rely on the cooperation and compliance of livestock farmers. It is unclear whether there is consensus in the industry on the strategy that should be followed to manage the disease. The objective of this study was to collect, assess, and summarize the views and opinions of stakeholders representing the DR swine production value chain on the alternative strategies that may be implemented to control ASF in the country. These views and opinions were organized into positive and negative attributes for each of the alternative strategies and presented to the DR government. Results presented here will contribute to building the conditions necessary to reach a consensus between the private and public sector on the expected impact and choice of alternative control strategies, with the ultimate goal of supporting the control of ASF in the country.

## SWOT analysis methodology

Snowball sampling was used to identify relevant experts for invitation into the study. In snowball sampling, relevant stakeholders are asked to identify individuals that they would consider experts for the relevant topic; thus, final expert selection was conducted by existing, private swine networks in the DR and not by the authors. Here, the initial, relevant stakeholders were considered as those representing the three sectors of the commercial swine production value chain in the DR. Namely, these sectors were pre-harvest, technical support, and post-harvest. To reach these stakeholders, beginning in February 2022, relevant swine industry organizations representing the three sectors were contacted and asked to provide a list of individuals they would consider as experts to respond to questions on ASF control. These organizations were the Federacion Dominicana De Porcicultores (FEDOPORC) and Asociacion Dominicana de Granjas Porcinas (ADOGRANJA), the two organizations representing swine producers (pre-harvest), the DR association of veterinarians and pharmaceutical companies (technical support), and the associations of pork product processors (post-harvest). No organizations exist that represent small-scale, backyard swine producers in the DR, and consequently that sector could not be surveyed through the methods used in this study. From these private stakeholders, a list of 14 experts was identified and invited to participate in the study. These experts were swine veterinarians, commercial swine producers, and swine processors/packers.

To capture the experts' opinions, a Strengths, Weaknesses, Opportunities, and Threats (SWOT) analysis was conducted. SWOT is a structured, analytical approach to assessing the advantages and disadvantages of a program (26). Strengths and weaknesses represent internal positive and negative attributes of a control program that facilitate or hinder its success. Internal attributes were described to participants as factors associated with planned control activities, the disease, and with the Dominican swine industry. Similarly, opportunities and threats are external or environmental factors that may help or hinder the program. External attributes were described as factors such as the Dominican government, culture, and society, and the international epidemiological context. SWOT has previously been used to assess health control programs in human and veterinary medicine (27, 28). For this analysis, three hypothetical scenarios of control were developed for consideration by experts: (1) total depopulation of all swine in the DR, (2) partial depopulation, and (3) continuation of current control measures. These scenarios were proposed because they represented alternative extremes and an intermediate level of ASF control and would be broad enough to allow for a wide range of responses from participants of varying expertise. Specific criteria for each scenario were not used to avoid artificially limiting the potential responses. Likewise, specific guidelines for partial depopulation were not proposed; instead, the scenario was described as one where a majority of farms would be depopulated, but those that committed to following certain surveillance, testing, and biosecurity standards would be allowed to remain in production. Continuation of current control measures included keeping surveillance and depopulation as currently implemented in the country.

Each expert was sent the SWOT survey *via* e-mail with follow-up *via* e-mail and phone calls to encourage participation and provide guidance in responding to the survey. The survey asked the experts identified *via* snowball sampling to consider the strengths, weaknesses, opportunities, and threats of each scenario in an open-question format. Following the SWOT analysis portion of the survey, additional questions were posed to the experts that considered aspects of biosecurity, surveillance, sampling, movement restrictions, border control with Haiti, vaccine use, wild boar and feral pigs, feed, and continuity of business. These questions varied between multiple-choice to open-question format. Demographic information including role in the swine industry, farming background, and years of experience were also collected. Prior to recruitment of experts, the survey and protocol were submitted to the University of Minnesota Internal Review Board and were given an exemption as non-human research. A total of 5 responses, representing the three sectors of the value chain, were collected. The responses were deidentified and compiled into one document for review. The opinions were summarized into one document of advantages and disadvantages for each scenario. For further confirmation of the analysis, part of the group and authors convened in-person in May 2022 to review the summary document and provide additional feedback. The resulting draft document was shared with the representatives of the swine value chain groups originally contacted and with the 14 designated experts. Finally, the results were presented to DR government officers for comments and input.

## SWOT findings

Based on our analysis, the key points from the SWOT analysis are summarized in Table 1. No clear consensus was apparent about the best strategy for ASF control in the DR and no single scenario was identified as ideal, but important positive and negative attributes (referred to as advantages and disadvantages, respectively) of each scenario were discussed and compared.

**Table 1:** Summarized responses on the strengths, weaknesses, opportunities, and threats of each ASF control scenario for the DR.

**Scenario**	**Advantages (strengths and opportunities)**	**Disadvantages (weaknesses and threats)**
**Scenario 1: Total depopulation**		
In this scenario, all domestic pigs (backyard production and commercial farms) in the Dominican Republic would be depopulated, with no exceptions.	More effective, fast, and safe eradication, achieving recertification as an ASF-free country in the shortest possible time. Previous experience carrying out a total eradication in the country. Opportunity to create a more organized and pathogen-free pig industry, including the elimination of CSF and PRRSV, and for the development of the meat import business. Offers of international technical and economic support for the program and responding to the regional concern about the ASF and CSF status of the DR.	Total eradication can fail for several reasons, including lack of sufficient funds, not knowing the location of backyard pigs, permissive controls, resistance from producers, lack of consensus, or insufficient political power to implement. Even if it is successful, the risk of new introduction from Haiti or other infected regions may result in a strategy of high economic and social cost that fails to sustain ASF-freedom. Social and economic impacts including market shortages, loss of the fresh pork market, labor unemployment, inoperative dead investments, food insecurity, disappearance of the informal trade of pork products such as chicharrones, roast pigs and artisanal sausages (mondongueros), and bankruptcy of producers.
**Scenario 2: Partial depopulation**		
In this scenario, most pig farms would be depopulated. However, farms that commit to following established standards, such as surveillance, intensive testing and biosecurity, would not be depopulated and would be allowed to remain in production.	Better control of the pig population and its health and productive status. Elimination of the informal population, which is the one that is perceived to increase risk. Maintain a pig industry for repopulation, creating ASF-free zones and maintaining the fresh meat market, level of production and labor. Possibility of evaluating a vaccine.	Difficult implementation (unknown location of backyard farms, constant repopulation, insufficient controls). ASF can become endemic, and outbreaks may recur. Longer recovery time. Greater financial impact for the state in the long run due to additional resources and time to eradicate ASF. Exports are still prohibited and less trust and support from the international community. Lack of unity among producers as some will benefit and others will not. Creation of conflict since imposing restrictions would form debates as to why one farm continues, and another would not. Conflicts between affected producers who do and do not comply with the rules would increase.
**Scenario 3: Continue with current control measures**		
African swine fever control strategies, such as surveillance and depopulation, would continue as currently carried out, with the potential for endemicity of African swine fever.	The Dominican population would continue to receive porcine protein of the highest quality. The government will not have to invest in funds for depopulation, disposal, or indemnity. Efficiency, health status and profitability improve for those who survive.	No examples of current control successes, in Europe or Asia, that can be replicated. Continued trade of infected animals, increasing the likelihood of outbreaks and endemicity that will destroy the industry. Investments in the industry would be very risky. Smaller scale, higher risk investments would lead to decreases in food safety and quality. International bodies and trading partners will eventually withdraw their support.

### Scenario 1: Total depopulation

Reported strengths of a depopulation program included a rapid and nearly certain way to eradicate ASF from the country, allowing for a more rapid recertification of disease freedom relative to other scenarios. A repeatedly cited opportunity associated with this strategy was the potential to eliminate other diseases prevalent in the DR, such as Classical swine fever (CSF) and porcine reproductive and respiratory syndrome (PRRS), and repopulate with a more organized, disease-free industry that would increase access to international markets. Additionally, the DR has previous experience with eradication (although it was acknowledged that conditions may have changed since that earlier experience 40 years ago). With ongoing international support due to concern about further disease spread, opportunities for outside resources to assist with this option were identified. However, many weaknesses and threats were highlighted that would undermine both the short and long-term success of total depopulation. Inconsistent tracing and incomplete knowledge of farm locations, especially backyard farms, currently impedes identification and culling of infected farms. This could make total depopulation difficult or nearly impossible to implement. Similarly, lack of sufficient funds for compensation for depopulated animals and insufficient political power to implement the program would also hinder its success. Many social and economic concerns were reported, such as unemployment, loss of fresh pork and market shortages, loss of traditional pork products, and financial losses or bankruptcy for producers. These factors may drive some producers to not comply with control strategies or hide their swine from animal health officials, and the market impact from depopulation could lead to financial and food insecurity for the general public. In summary, many experts were concerned that total depopulation might lead to long-term or permanent disruption of the DR pork industry with negative effects to non-swine sectors. Even if total depopulation were carried out, ASF reintroduction remains a viable threat. Limited border control and a weak political situation in Haiti, where ASF outbreaks are also ongoing, could lead to ASF reintroduction soon after repopulation.

### Scenario 2: Partial depopulation

A reported major strength of this strategy was the development of long-term disease control within the swine industry through elimination of populations that could potentially increase disease risk, such as those with limited biosecurity. Compared to total depopulation, this scenario would leave the swine industry with a healthy population of pigs to support repopulation, allowing a quicker return to production. This would also allow for the country to maintain a certain level of pork production and labor, lessening the overall financial burden and market disruption. Maintaining some production may also provide the opportunity to evaluate an ASF vaccine and potentially have early access to vaccines for future control strategies. Similar to scenario 1, concerns about the ability to locate pig farms for depopulation, such as smallholders or backyard producers that may be unknown to government officials, were reported. Unidentified farms could act as virus reservoirs and maintain endemicity, leading to future outbreaks and ultimately more time and resources spent by the government to eradicate ASF. Additionally, though the market impact of partial depopulation was predicted to be less than scenario 1, losses would accrue until the industry returned to full production levels. Many international markets would also likely remain inaccessible due to concerns about the ASF-status in the DR. Social threats were also identified, including conflicts between producers due to rules or criteria used to determine whether a farm would be depopulated or allowed to remain in production. This potential perceived inequality could undermine overall efforts for ASF control and lead to social disruption.

### Scenario 3: Continue with current control measures

Fewer strengths and opportunities were listed for this scenario, but those reported mainly consisted of maintaining the current availability of high-quality pork and requiring minimal financial investment from the government. Weaknesses and threats reflected concerns about the ongoing situation: continuation of local trade of infected animals leading to disease endemicity and further disruption to the DR pork industry, with long-term investment perceived as increasingly risky. Consequently, it was suggested that this scenario will result in lower investment from the private sector, gradually affecting the quality of the product and eventually hampering the DR swine industry as a whole. Another concern was the lack of examples of successful control strategies.

## Discussion

The study highlighted the difficulty in choosing any one particular control strategy for ASF in the DR, with no particular strengths and opportunities seeming to outweigh their corresponding weaknesses and threats, or those of the other scenarios. Important concerns were raised for each scenario that are common to ASF control globally, such as social and economic impact of total or mass depopulation, food security, animal welfare, and the ability of governments to implement controls with limited power, veterinary infrastructure, or resources (Table 1).

A common theme to the weaknesses and threats of each scenario was balance between competing factors, including economically and socially. While animal health officials and epidemiologists may be focused on total eradication of ASF, livestock producers often compare its impact to ongoing endemic disease control, market impact, costs to implement surveillance and biosecurity, and other concerns. For example, mass depopulation from an ASF introduction may mean the elimination of a producer's livelihood, and they may favor less severe control measures, especially if they consider other diseases such as PRRS to be more impactful than ASF. This context is critical to understand, as without the participation of the private swine industry, official control efforts are likely to fail. Even worse for ASF control, some producers may react to control measures by hiding livestock or using unmonitored markets to sell pigs.

As the SWOT results suggested, ASF control may be a particular concern for backyard or small-holder producers in the DR (Table 1). As no formal registry of these producers exists, this assessment primarily represents the opinion of the formal swine industry in DR. Despite the lack of their explicit inclusion, the perceptions captured here consider DR swine production as a whole, including the impact to and role of backyard producers, when considering alternative ASF control strategies. For example, partial depopulation may protect the industry, but it likely favors large producers who can invest in biosecurity or who have the financial resources to survive until they can repopulate, while small or backyard producers may be largely eliminated from the industry altogether. These losses damage families and communities that rely on swine for subsistence, financial support, and where swine play important cultural roles. These impacts must be balanced against the need to prevent widespread ASF transmission. Similar situations play out in many affected countries, where governments and animal health officials must balance competing concerns in different epidemiological, economic, and social contexts (13, 14, 29–32).

A repeatedly suggested opportunity from the SWOT analysis was to use the current ASF situation to improve the DR swine industry as a whole (Table 1). Many of the reported weaknesses and threats in each proposed control strategy are in part due to the lack of or inconsistent infrastructure and resources available to both public and private stakeholders. The increased attention and resources available internationally for controlling ASF might be used improve factors in the DR such as traceability of swine farms and movements, biosecurity, and surveillance support. This would not only reduce disease burden from ASF but also improve long-term management of endemic diseases like PRRS and CSF. Developing control strategies in this holistic way may also incentivize participation from private swine farms and overall improve the strategies' success.

Transparency and teamwork between the public and private swine sectors may help to bridge gaps between these stakeholders and support more sustainable, successful control strategies in the future. This study documents an approach that stakeholders can use to improve these partnerships, and thereby engagement in mitigation and control efforts for ASF and other transboundary diseases. These results also demonstrate that SWOT analyses are a useful tool in veterinary medicine to assess alternative control strategies and connect private and public stakeholders. Here, following the review with private stakeholders, a summary of the results was presented to officials from the DR government. This prompted further in-depth discussion to reflect on the private opinions that were gathered and was valuable for ASF control planning. These types of analyses also allow researchers to act as moderators in the discussion, bringing together stakeholders that might otherwise not meet, and for ideas to be shared openly from all perspectives. This is a highly valuable tool for disease planning and should be implemented to help create and maintain public-private partnerships. Future efforts to control ASF in the DR should include the continuation of these discussions and inclusion of the different sectors of the DR swine industry in planning.

Ultimately, the challenges identified here have likely contributed to ASF's endemicity in many regions. In a broader sense, the results also improve our understanding of the reasons behind challenges associated with ASF control and suggest the need to explore novel approaches when attempting to control the animal disease that, arguably, has spread most widely globally in recent years.

## Data availability statement

The original contributions presented in the study are included in the article/supplementary material, further inquiries can be directed to the corresponding author/s.

## Author contributions

AP and RR designed the study. RS created the questionnaire and all authors reviewed it. RR, JM, and AD distributed and collected questionnaires. RS and AP reviewed responses and performed the analysis. RS wrote the manuscript with input and review from all authors. All authors participated in presentation of findings to the expert group. All authors contributed to the article and approved the submitted version.

## Funding

This research was supported in part by the U.S. Department of Agriculture (USDA), Animal and Plant Health Inspection Service. The findings and conclusions in this publication are those of the author(s) and should not be construed to represent any official USDA or U.S. Government determination or policy.

## Conflict of interest

The authors declare that the research was conducted in the absence of any commercial or financial relationships that could be construed as a potential conflict of interest.

## Publisher's note

All claims expressed in this article are solely those of the authors and do not necessarily represent those of their affiliated organizations, or those of the publisher, the editors and the reviewers. Any product that may be evaluated in this article, or claim that may be made by its manufacturer, is not guaranteed or endorsed by the publisher.
